# An experimental study of the effect of anxiety on lexical processing of college students: evidence from true-false word judgment and semantic category judgment tasks

**DOI:** 10.3389/fpsyg.2025.1452867

**Published:** 2025-03-13

**Authors:** Zhang Huiyong, Pu Xinping

**Affiliations:** ^1^Student Mental Health Education Center of Tiangong University, Tianjin, China; ^2^Key Research Base of Humanities and Social Sciences of the Ministry of Education, Academy of Psychology and Behavior, Tianjin Normal University, Tianjin, China; ^3^Faculty of Psychology, Tianjin Normal University, Tianjin, China; ^4^Tianjin Key Laboratory of Student Mental Health and Intelligence Assessment, Tianjin, China

**Keywords:** anxiety, true-false word judgments, semantic categories, lexical processing, college students

## Abstract

**Introduction:**

This study examined the effect of anxiety on lexical processing among college students with anxiety in China.

**Methods:**

We conducted two experiments in a stressful environment to induce anxiety. Experiment 1 investigated the effect of anxiety on lexical processing through a true-false word judgment task, while Experiment 2 further explored this effect using a semantic category judgment task.

**Results:**

Both experiments revealed no significant difference in the accuracy of lexical judgments between participants with high and low anxiety. However, there was a notable difference in the reaction times for lexical judgments, with high-anxiety participants exhibiting longer reaction time compared to their low-anxiety counterparts. This indicates a decrease in the efficiency of lexical processing among those with high anxiety.

**Conclusion:**

This study confirms that anxiety diminishes lexical processing efficiency without affecting lexical judgment performance. These findings support the processing efficiency theory.

## 1 Introduction

Anxiety is a future-oriented, long-term response primarily focused on vague threats (Vandebos, 2007). It is often accompanied by increased physiological arousal and somatic tension. Persistent and excessive anxiety can lead to both physical and psychological harm, potentially developing into an anxiety disorder. Previous research has demonstrated that high levels of anxiety impair cognitive processing efficiency (Berggren and Derakshan, 2013; Derakshan and Eysenck, 2009; Eysenck and Calvo, 1992; Eysenck et al., 2007; Humphreys and Revelle, 1984). According to the processing efficiency theory (Eysenck and Calvo, 1992), anxiety negatively impacts cognitive processing, which encompasses both processing efficiency and operational performance. The theory posits that anxiety has a more significant impact on processing efficiency than on operational performance. Furthermore, anxiety hinders the central executive function within the working memory system. This is evidenced by the fact that anxiety consumes a substantial amount of working memory resources and necessitates additional time for information processing to mitigate its negative effects on cognitive processing, ultimately leading to a decline in processing efficiency. Lexical processing is also a cognitive task, particularly in reading, where it serves as the fundamental unit of reading comprehension. This study examines the influence of anxiety on lexical processing through a true-false word judgment task and a semantic category judgment task.

Previous research has shown that high-anxiety readers require more cognitive processing resources to comprehend words in text. In stressful situations, these readers tend to spend more time on reading tasks and demonstrate lower efficiency compared to low-anxiety readers; however, their reading comprehension scores remain unaffected (Calvo et al., 1992; Calvo and Carreiras, 1993; Calvo et al., 1994b; Calvo, 1996). These findings align with processing efficiency theory (Eysenck and Calvo, 1992), which suggests that individuals with high anxiety need additional processing resources and take longer to process information than their low-anxiety counterparts. Consequently, the greater the cognitive resources demanded by a reading task, the more pronounced the negative impact of anxiety on efficiency, as high-anxiety readers must allocate extra time to compensate for the working memory resources consumed by their anxiety. Zhao et al. (2022, 2023) examined the role of working memory capacity in the relationship between anxiety and interpretation. The study found that both greater working memory capacity and lower levels of anxiety contributed to improved interpreting performance. This suggests that the relationship between anxiety and reading comprehension is influenced by working memory capacity.

How to effectively trigger anxiety is an important question when examining its impact on lexical processing. Research has demonstrated that trait anxiety reduces the efficiency of cognitive task processing under situational stress, and that it is associated with a decrease in processing efficiency in specific stressful situations (Edwards et al., 2016; Morris et al., 1981; Sorg and Whitney, 1992). MacIntyre and Gardner’s (1994) study investigated the arousal of anxiety triggered by a video camera at various stages of a lexical learning task. They found that the presence of a video camera significantly increased participants’ anxiety levels, which in turn led to observable deficits in lexical acquisition. We employed this current method of anxiety elicitation based on the findings of this previous literature.

Given that lexical elements are the fundamental components that constitute sentences and texts, lexical recognition is essential for reading comprehension and semantic integration. However, there has been limited research on the impact of anxiety on lexical processing in isolation, particularly studies that focus solely on how anxiety affects the efficiency of lexical processing. Therefore, the present study aimed to investigate the effect of anxiety on lexical processing efficiency by creating stressful situations. This was achieved by using a video camera to induce higher levels of anxiety in participants, through two experiments: a true-false word judgment task and a semantic category judgment task.

## 2 Experiment 1: the effect of anxiety on a true-false word judgment task

### 2.1 Methods

#### 2.1.1 Participants

The Chinese version of the Trait Anxiety Inventory (TAI) of the State-Trait Anxiety Inventory (STAI), translated and revised by Li and Qian (1995), was used to screen participants for trait anxiety in this study. A total of 2,189 assessments were distributed via the Questionnaire Star platform. Of these, 2,039 valid questionnaires were retrieved, with a validity of 93.15%. The sample comprised 1,418 men and 621 women, aged 18–24 years, with a mean age (M) of 20.22 years and a standard deviation (SD) of 1.07. Using a criterion of a score greater than 55 on the High Trait Anxiety Questionnaire, 27 participants were selected in descending order of TAI scores for the High Trait Anxiety group, with ages ranging from 18 to 24 years (M = 19.07, SD = 1.84) and TAI scores (M = 58.19, SD = 5.26). Conversely, for the Low Trait Anxiety group, participants with scores below 30 were selected in ascending order, and 26 participants were selected in the trait anxiety group, aged 18 to 25 years (M = 19.88, SD = 5.25) with TAI scores (M = 22.50, SD = 1.30). The age difference between the two groups of participants was not statistically significant (*t*_(51)_ = 0.98, *p* = 0.33), while the difference in TAI scores was statistically significant (*t*_(51)_ = 33.59, *p* < 0.001). A total of 53 college students with high and low trait anxiety were selected, and all participants had normal or corrected-to-normal vision. Prior to the experiment, each participant signed an informed consent form, indicating that their participation was voluntary, and they received 20 RMB as compensation. This study was approved by the Ethics Committee of Tianjin Normal University (approval number: 22JJD190012).

#### 2.1.2 Stressful situation setting

This study identified participants with high and low trait anxiety based on prior research (Meijer, 2001; Spielberger et al., 2015) and induced levels of anxiety by creating stressful situations, following the methodology outlined by Calvo et al. (1994a, 1994b). Initially, a video camera was positioned in front of each participant; the presence of the camera served as a stressor (Otto, 1990). Subsequently, a form of self-threat was introduced. This self-threat, also known as evaluative stress, is widely employed in anxiety performance studies and represents a common method for stress manipulation. It involves participants facing adverse evaluations of their performance (Leary et al., 2009), which can provoke anxiety, worry, and other detrimental effects (Edwards et al., 2015; Leary et al., 2009; Moran, 2016). In this study, participants were informed that the entire experiment would be videotaped and that the results of their lexical judgments would be evaluated by the experimenter and that their results would be compared with those of other students, which could be stressful and lead to participants feeling that they were being judged unfavourably. Finally, each participant’s name was recorded after they had been assured that their results would be kept confidential.

#### 2.1.3 Materials

Based on previous studies (Allen and Emerson, 1991; Allen et al., 1995), a total of 360 stimuli (120 true words, 120 false words, and 120 filler words) were used. Among them, the false words were constructed using two true characters of Chinese characters. All experimental words were randomly mixed, ensuring that each participant received the complete set of experimental materials.

#### 2.1.4 Design

A one-way experimental design was used. The independent variables were high and low anxiety and the dependent variables were accuracy and reaction time.

#### 2.1.5 Procedure

The experimental procedure was programmed using E-Prime 3.0 and was conducted as follows: (1) participants read the instructions; (2) after understanding the experimental procedure, they engaged in practice items, which consisted of a total of 20 practice words. Judgments on each practice word were provided along with feedback on correctness and errors, as well as reaction time to help participants prepare for the formal experiment; (3) the experiment began with the presentation of a red “+” dot in the center of the screen for 500 ms, indicating that the target stimulus was about to appear, followed by the presentation of the target stimulus in the center of the screen; (4) participants made a true-false judgment about the presented stimulus (based on the criterion of whether or not two Chinese characters make up an intelligible word), which was displayed for 2,000 ms. If the word was true, participants pressed the “F” key; if it was false, they pressed the “J” key (key presses were based on rapid responses with hand dominance).

**Figure 1:**
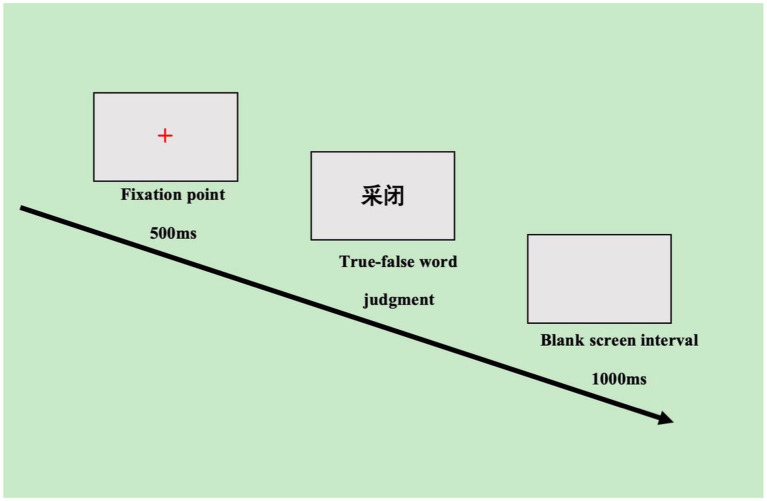
Flowchart of true-false word judgment task.

To provide participants time to prepare for the next stimulus, the presentation interval for the target stimulus was set at1,000 ms. Additionally, there were five evenly spaced breaks, each lasting 3 min, throughout the experiment (see Figure 1).

### 2.2 Results

Data were organized prior to formal data analysis by removing data with error rate higher than 15% and removing extreme data greater than three standard deviations, which accounted for 2.7% of the valid participant data. Statistical processing of the accuracy and reaction time data was performed using SPSS 22.0 software.

The accuracy and reaction time of true-false word judgments of high-anxiety and low-anxiety participants are shown in Table 1. The results show that the difference between high anxiety and low anxiety on the accuracy of neutral word judgment is not significant (*t*_(48)_ = −1.71, *p* = 0.09). The reaction time of high-anxiety individuals is significantly longer than the reaction time of low-anxiety individuals (*t*_(48)_ = 3.15, *p* < 0.01).

**Table 1:** Descriptive statistics of accuracy and reaction time for true-false judgements of words (M ± SD).

Participant type	Accuracy (*M* ± *SD*)	Reaction time (*M* ± *SD*)
High anxiety	0.96 ± 0.19	713 ± 227
Low anxiety	0.98 ± 0.15	682 ± 211

Experiment 1 employed a true-false word judgment task to investigate the efficiency of lexical processing among anxious college students in a stressful situation. The results showed that there was no significant difference in the correctness of true-false word judgments between high-anxiety and low-anxiety college students. However, there was a significant difference in the reaction time for these judgments; high-anxiety college students exhibited slower reaction time compared to their low-anxiety counterparts. This finding aligns with the theoretical hypothesis of processing efficiency, which suggests that anxiety consumes a substantial amount of working memory resources. Consequently, more time is required for information processing to reduce the negative effects of anxiety on lexical processing, leading to less efficient lexical processing overall.

Unlike true-false word judgments, semantic category judgments require participants to extract meaning, providing more direct evidence for examining the effect of anxiety on semantic processing. In the true-false word judgment task, participants may not need to fully understand the semantics to make a decision. Experiment 2 offers a more comprehensive analysis at the semantic processing level to further validate the effect of anxiety on lexical processing efficiency.

## 3 Experiment 2: the effect of anxiety on a semantic category judgment task

### 3.1 Methods

#### 3.1.1 Participants

Participants and situational stress settings were the same as in Experiment 1.

#### 3.1.2 Materials

According to previous research (Chen et al., 2007), 90 two-character words were selected that semantically belong to the category of “action.” An additional 90 two-character words that do not semantically belong to the action category were used as fillers. The typicality of the vocabulary within the action category was evaluated. Thirty subjects, who did not participate in the formal experiment, rated the typicality of 90 selected two-character words in this category. A 7-point scale was employed, where 1 indicates that a word does not belong to the semantic category of action at all, and 7 signifies that it typically belongs to this category, reflecting an increasing degree of semantic association with the action category. The results show that the mean typicality rating for the words in the action category is 6.10, indicating that all selected vocabulary items are considered typical within this category.

#### 3.1.3 Design

A one-way experimental design was used in the context of semantic category judgments. The independent variables included high and low anxiety, while the dependent variables consisted of accuracy (the rate of correctly identifying whether a word belongs to a semantic category) and reaction time (the duration taken to accurately determine whether a word is a semantic category word).

#### 3.1.4 Procedure

The experimental procedure was programmed using E-Prime 3.0 and was conducted as follows: (1) participants read the instructions; (2) after understanding the experimental procedure, they engaged in practice items, which consisted of a total of 20 practice words. Judgments on each practice word were provided along with feedback on correctness and errors, as well as reaction time to help participants prepare for the formal experiment; (3) at the beginning of the experiment, a 500-ms “+” dot was presented in the center of the computer screen to indicate that a target word was about to appear. After the dot disappeared, the target word was displayed in the same position for a duration of 2,000 ms. (4) Participants were asked to judge whether the meaning of the target word displayed in the center of the screen belonged to the semantic category (criteria based on whether or not the semantics of the word belong to the semantic category of “action,” e.g., “playing ball”) of “F” key was pressed if the word fell within the action category, while the “J” key was pressed if it did not (key presses were based on rapid responses with hand dominance).

**Figure 2:**
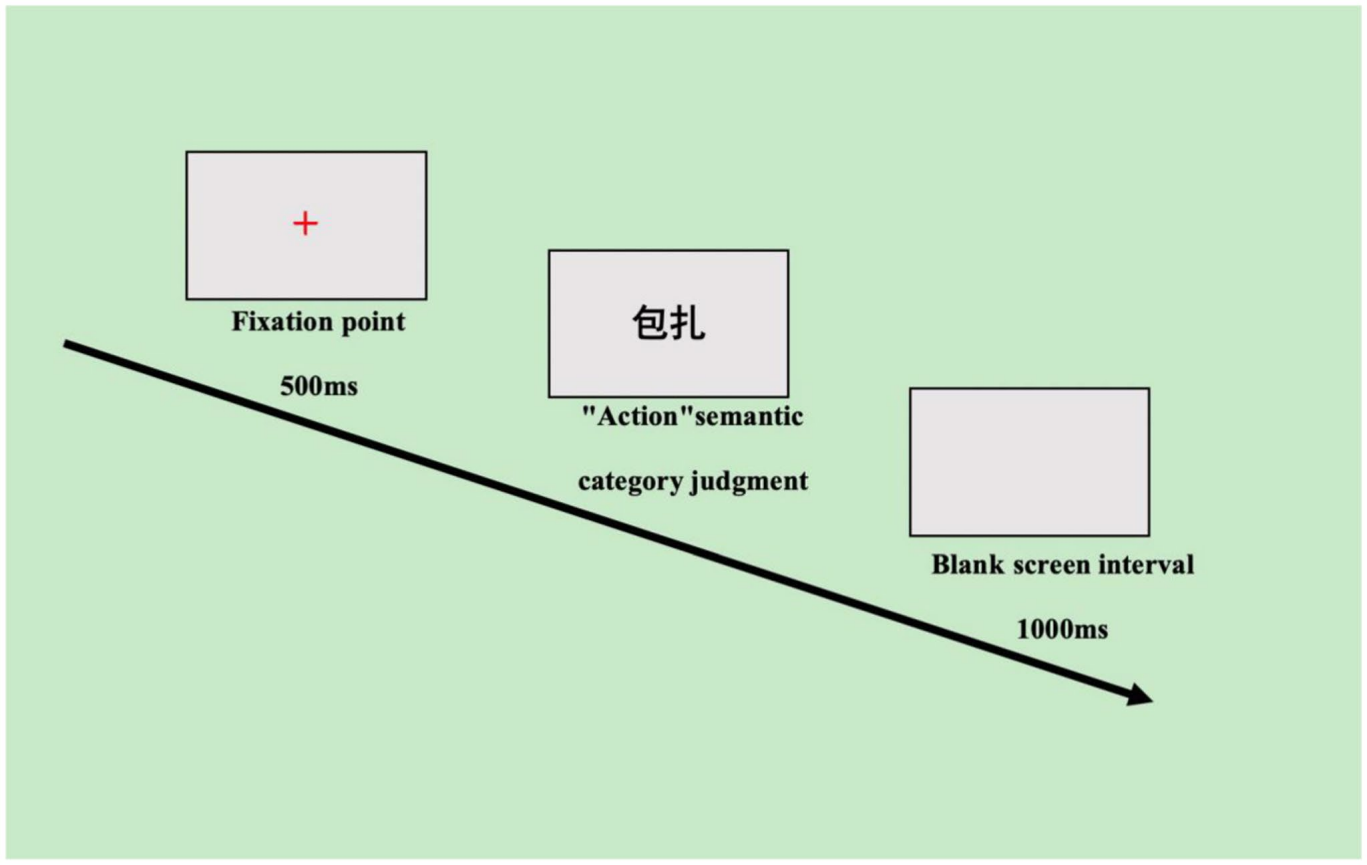
Flowchart of the semantic category judgment task.

To allow participants sufficient time to prepare for the next stimulus, the presentation interval for the target stimulus was set at 1,000 ms. Additionally, there were five evenly spaced breaks, each lasting 3 min, throughout the experiment (see Figure 2).

### 3.2 Results

Data were organized prior to formal data analysis by removing data with error rate higher than 15% and removing extreme data greater than three standard deviations, which accounted for 0.8% of the valid participant data. Statistical processing of the accuracy rate and reaction time data was performed using SPSS 22.0 software.

The accuracy and reaction time of high-anxiety and low-anxiety participants on lexical semantic category judgments are shown in Table 2. The results show that the difference between the accuracy of lexical semantic category judgment between high and low anxiety is not significant (*t*_(48)_ = 1.6, *p* = 0.11). The reaction time of lexical semantic category judgment of high-anxiety individuals is significantly longer than the reaction time of low-anxiety individuals (*t*_(48)_ = 24.78, *p* < 0.001).

**Table 2:** Descriptive statistics of accuracy and reaction time for lexical semantic category judgements (M ± SD).

Participant type	Accuracy (*M* ± *SD*)	Reaction time (*M* ± *SD*)
High anxiety	0.97 ± 0.16	722 ± 243
Low anxiety	0.97 ± 0.18	567 ± 134

In Experiment 2, a stressful situation was created, and a semantic category judgment task was used to further examine the effect of anxiety on lexical processing efficiency. The results indicated that there was no significant difference in the accuracy of semantic category judgments between college students with high anxiety and those with low anxiety. However, there was a significant difference in the reaction times for semantic category judgments; students with high anxiety exhibited slower reaction time compared to their low-anxiety counterparts. This finding suggests that anxiety adversely affects lexical processing efficiency more than it does lexical processing accuracy.

The experimental results further support the processing efficiency theory by showing that anxiety requires more time to reduce the negative effects on lexical processing. This leads to less efficient lexical processing overall. Furthermore, the findings indicate that anxiety has a greater effect on lexical processing efficiency than on lexical judgment scores.

## 4 Discussion

Previous research on the effect of anxiety on reading has primarily focused on textual analysis. However, few studies have investigated the effect of anxiety on lexical processing in isolation. This study examined the influence of anxiety on lexical processing through two experiments. The results found that anxiety did not significantly affect the accuracy of lexical judgments; instead, it led to a decrease in the efficiency of lexical processing. This inefficiency was primarily observed in the longer time taken by participants with high anxiety to make lexical judgments compared to those with low anxiety. This finding will be discussed in the following three aspects:

First, anxiety reduced the efficiency of lexical processing. Both Experiments 1 and 2 examined the effect of anxiety on lexical processing at the lexical level, excluding the effect of contextual and syntactic information. In Experiment 1, participants with high anxiety had significantly longer reaction times to true-false word judgments than participants with low anxiety. Experiment 2 also used a semantic category judgment task, in which semantics had to be communicated in order to make accuracy judgments, and again found that high-anxiety participants’ lexical processing took longer. The results of both experiments suggest that anxiety affects lexical processing, leading to a decrease in processing efficiency. It is worth noting that the semantic category judgment in Experiment 2 was made on the basis of extracting semantics, which provides important evidence for investigating the effect of anxiety on lexical processing and lays the foundation for further research investigating the effect of anxiety on sentence processing.

Second, this study supported the hypotheses of processing efficiency theory (Eysenck and Calvo, 1992). The processing efficiency theory suggests that anxiety has a greater effect on processing efficiency than on operational performance, that anxiety depletes working memory resources, and that high-anxiety individuals need more time to compensate for the negative effect of anxiety on processing efficiency. The present study found that anxiety did not affect performance on lexical judgments, but did reduce the efficiency of lexical processing. Through Experiments 1 and 2, it was found that there was no significant difference in the accuracy of lexical judgments between the high-anxiety group and the low-anxiety group, indicating that anxiety had no effect on lexical comprehension. On the other hand, the lexical judgments of the high-anxiety group took longer and the efficiency of lexical processing was lower, indicating that the high-anxiety group needed more time to compensate for the negative effect of anxiety on the efficiency of lexical processing during the lexical processing process. The results of the two experiments suggest that anxiety affects lexical processing and leads to a decrease in processing efficiency.

Third, the mechanism by which anxiety affects the efficiency of lexical processing may be related to working memory. Zhao’s et al. (2023) study found that the interpreting performance of anxious individuals would be related to their working memory capacity, and that subjects with greater working memory capacity had higher interpreting performance. The present study found that the lexical processing of highly anxious subjects took longer and was less efficient, which may also be related to their working memory capacity. The processing efficiency theory suggests that anxiety reduces working memory capacity, leading to a decrease in processing efficiency. Highly anxious people may have less working memory capacity, and less working memory capacity leads to less efficient lexical processing. Although we did not test the subjects’ working memory capacity, we believe that it influences the effect of anxiety on lexical processing efficiency, and that working memory capacity may also be causally related to the relationship between anxiety and reading efficiency, which we have not yet investigated. In future studies, we have manipulated working memory as an independent variable in our experiment to further explore the role of working memory in the effect of anxiety on lexical processing efficiency.

Our study highlights that higher anxiety reduces the efficiency of lexical processing, which is the most basic unit of reading. Therefore, higher anxiety also reduces the reading and learning efficiency of college students. Given that mindfulness meditation significantly reduces the anxiety level of college students (Bamber and Schneider, 2016; Bamber and Erin, 2018), implementing this approach with high-anxiety college students will improve their reading and learning efficiency. Intervention research and training for high-anxiety college student readers could also be conducted in the future. From the perspective of psychological intervention, effective mindfulness training and intervention for high-anxiety college students will improve their reading and learning efficiency.

## 5 Conclusion

This study verified the theoretical view of processing efficiency through two experiments. The following conclusions can be drawn: first, the lexical processing efficiency of high-anxiety college students decreases, which is manifested by the fact that there is no difference between high-anxiety and low-anxiety college students in the scores of lexical judgments, but in the reaction time of lexical judgments, the high-anxiety college students are significantly slower than the lexical processing time of low-anxiety college students, indicating that anxiety reduces the efficiency of lexical processing; second, the decrease in lexical processing efficiency of high-anxiety college students is also related to the depth of their lexical extraction, which is manifested in the fact that the reaction time of high-anxiety college students for semantic category judgments is slower than that of low-anxiety college students, and the efficiency of extracting semantic information is lower. Future findings on the differential effects of anxiety on lexical processing efficiency in more anxious populations, such as adolescents, can also be explored in depth, which may further validate the generalisability of the findings, helping teachers to better target the learning efficiency of high-anxiety students, thus providing practical guidance for high-anxiety students to successfully pass exams and complete their studies.

## Data Availability

The original contributions presented in the study are included in the article/supplementary material, further inquiries can be directed to the corresponding author.
